# *Sebekia* sp. (Eupentastomida, Sebekidae) in *Pygocentrus piraya* (Actinopterygii: Serrasalmidae) from marginal lagoons of the middle São Francisco River basin, Brazil

**DOI:** 10.1590/S1984-29612022060

**Published:** 2022-11-21

**Authors:** Rayane Duarte, Michelle Daniele dos Santos-Clapp, Marilia de Carvalho Brasil-Sato

**Affiliations:** 1 Programa de Pós-graduação em Ciências Veterinárias, Instituto de Veterinária, Universidade Federal Rural do Rio de Janeiro - UFRRJ, Seropédica, RJ, Brasil; 2 Laboratório de Biologia e Ecologia de Parasitos, Departamento de Biologia Animal, Instituto de Ciências Biológicas e da Saúde, Universidade Federal Rural do Rio de Janeiro - UFRRJ, Seropédica, RJ, Brasil

**Keywords:** Lagoon environment parasitology, lagoon fish parasites, Pentastomida, Porocephalida, Parasitologia de ambiente lagunar, parasitos de peixes de lagoas, Pentastomida, Porocephalida

## Abstract

*Pygocentrus piraya* is an endemic species of the São Francisco River basin. In this study, 342 carnivorous fish were examined, nymphs specimens of *Sebekia* sp. and its ecological indexes are recorded only in piranhas, of the total of 53 collected from three marginal lagoons of the middle São Francisco River basin: 17 specimens from Curral de Varas lagoon; 15 from Maris lagoon, both of state of Minas Gerais; and 21 specimens from Mocambo lagoon, state of Bahia. The prevalence (P) and mean abundance (MA) of *Sebekia* sp. nymphs in intermediate hosts were as follows: in Curral de Varas lagoon, P = 11.8%, MA = 0.18; in Maris lagoon, P = 46.6%, MA = 0.47; and in Mocambo lagoon, P = 14.3%, MA = 1.05. Despite the lower number of fish examined from Maris lagoon, this lagoon had the highest number of piranhas (seven) parasitized by *Sebekia* sp. Pentastomids have zoonotic importance and evaluation of the indirect life cycle of sebekids in lagoons is necessary for defining the intermediate and final hosts involved. This record is novel and stems from collection of piranhas in the marginal lagoons of the São Francisco River, in the states of Minas Gerais and Bahia, Brazil.

## Resumo

*Pygocentrus piraya* é espécie endêmica da bacia do rio São Francisco. Neste estudo, 342 peixes carnívoros foram examinados, espécimes ninfais de *Sebekia* sp. e seus índices ecológicos são registrados apenas em piranhas, do total de 53 coletadas em três lagoas marginais da bacia do médio rio São Francisco: 17 espécimes da lagoa Curral de Varas; 15 da lagoa Maris, ambas no estado de Minas Gerais; e 21 espécimes da lagoa Mocambo, estado da Bahia. A prevalência (P) e a abundância média (AM) das ninfas de *Sebekia* sp. nos hospedeiros intermediários foram: na lagoa Curral de Varas P = 11,8%, AM = 0,18; na lagoa Maris, P = 46,6%, AM = 0,47; e na lagoa Mocambo, P = 14,3%, AM = 1,05. A lagoa Maris, apesar do menor número de peixes examinados, foi a que apresentou maior número de piranhas parasitadas (sete) por *Sebekia* sp. Os pentastomídeos têm importância zoonótica, e a avaliação do ciclo de vida indireto dos sebekídeos em lagoas é necessária para definir os hospedeiros intermediários e finais envolvidos. Este registro é inédito e decorre da coleta de piranhas nas lagoas marginais do rio São Francisco, nos estados de Minas Gerais e Bahia, Brasil.

## Introduction

With diverse ichthyofauna, socioenvironmental importance, especially for fishing, the basin of the São Francisco River is the largest hydrographic basin in Brazilian territory (Godinho & Godinho, 2003). Among its fish fauna, the São Francisco piranha, *Pygocentrus piraya* (Cuvier, 1819) (Actinopterygii, Characiformes, Serrasalmidae), which was originally allocated in *Serrasalmus* Lacepède, 1803 (Fink, 1993), is an endemic species (Froese & Pauly, 2022). It has carnivorous feeding habits and is opportunistic (Britski et al., 1988).

The phylum Pentastomida comprises eight fossil species and 141 recent species and subspecies (Christoffersen & De Assis, 2015) and is a group of neglected endoparasites that parasitize several classes of vertebrates. These parasites have the potential to cause losses in aquaculture (Giesen et al., 2013) and pentastomiasis, including in humans (Mairena et al., 1989; Paré, 2008). Their morphology is complex and the various phylogenetic inferences that can be made about pentastomids indicate that they do not share convincing synapomorphies with any group within Arthropoda, but place them unquestionably among the Ecdysozoa. They undergo a series of molts until they reach the adult stage (Christoffersen & De Assis, 2015).

For adult specimens of the species of *Sebekia* Sambon, 1922 (Eupentastomida, Porocephalida, Porocephaloidea, Sebekidae), crocodilians are generally the definitive hosts. The nymphs of *Sebekia* spp. have been listed as endoparasites of species of various orders of fish in different water systems (Christoffersen & De Assis, 2013). Among these, *Pygocentrus nattereri* Kner, 1858 [as *Serrasalmus nattereri* (Kner)] (Characiformes, Serrasalmidae) inhabits the Pantanal region of the state of Mato Grosso, Brazil (Rego & Vicente, 1988). Some nymphs of sebekids, e.g. *Leiperia gracilis* (Diesing, 1836) Sambon, 1922 [as *Pentastomum gracile* Diesing, 1836] (Leiperiinae) and *Sebekia oxycephalum* (Diesing, 1836) Sambon, 1922 (Sebekinae), were recorded in fish originally referred to as "serrasalmo piranha" in the state of Mato Grosso, Brazil (Christoffersen & De Assis, 2013). This old vernacular nomenclature was used for both *P. nattereri* and *P*. *piraya,* and possibly referred to parasitism in *P. nattereri*, considering that its distribution includes the hydrographic basins that cover the state of Mato Grosso, while *P*. *piraya* is an endemic species in the São Francisco River basin (Froese & Pauly, 2022).

In *P. nattereri* in Brazil, Eiras et al. (2010) listed *Subtriquetra* sp., *L. gracile*, *Leiperia* sp. and *S. oxycephala*; Barros et al. (2010) recorded Pentastomida gen. sp. in hosts in the Cuiabá River, state of Mato Grosso; Vicentin et al. (2013) registered *Subtriquetra* sp._1_, *Subtriquetra* sp._2,_ *L*. *gracile* and *S*. *oxycephala*, in host piranhas in the Negro River, Pantanal, state of Mato Grosso do Sul; and Giesen et al. (2013) recorded *Subtriquetra subtriquetra* Sambon, 1922, *Leiperia* sp., *Alofia* sp. and *Sebekia* sp. in these piranhas in the Miranda River, Pantanal, state of Mato Grosso do Sul.

The aim of the current study was to register the occurrence of *Sebekia* sp. parasite of *P. piraya* from three lagoons bordering the middle São Francisco River, states of Minas Gerais and Bahia, Brazil.

## Materials and Methods

A total of 342 specimens of carnivorous fish (Actinopterygii, Characiformes), comprising 106 specimens of *Acestrorhynchus lacustris* (Lütken, 1875) (Acestrorhynchidae), 71 specimens of *Hoplias malabaricus* (Bloch, 1794) (Erythrinidae), 80 specimens of *P. piraya* and 85 specimens of *Serrasalmus brandtii* (Lütken, 1875) (Serrasalmidae), were provided with fractional collections between October 2007 and September 2018 by the management team of the project “Revitalization of the marginal lagoons of upper-middle São Francisco River basin, Minas Gerais, Brazil” for parasitological analysis. This project was conducted in cooperation between the Brazilian Institute for the Environment and Renewable Natural Resources (Instituto Brasileiro do Meio Ambiente e dos Recursos Naturais Renováveis, IBAMA) and the Development Company for the São Francisco and Parnaíba Valleys (Companhia de Desenvolvimento dos Vales do São Francisco e do Parnaíba, CODEVASF). These fish were fixed in 3% formalin, marked with biometric data and the lagoon name (on tags) and individually packaged inside plastic bags. They were then sent to the Parasite Ecology and Biology Laboratory (Laboratório de Biologia e Ecologia de Parasitos, LABEPAR) of the Federal Rural University of Rio de Janeiro (Universidade Federal Rural do Rio de Janeiro, UFRRJ), Seropédica, state of Rio de Janeiro, Brazil, where they were examined. Among these four species of piscivorous fish, only the specimens of *P. piraya* were parasitized by the pentastomids recorded in the present study.

**Figure 1:**
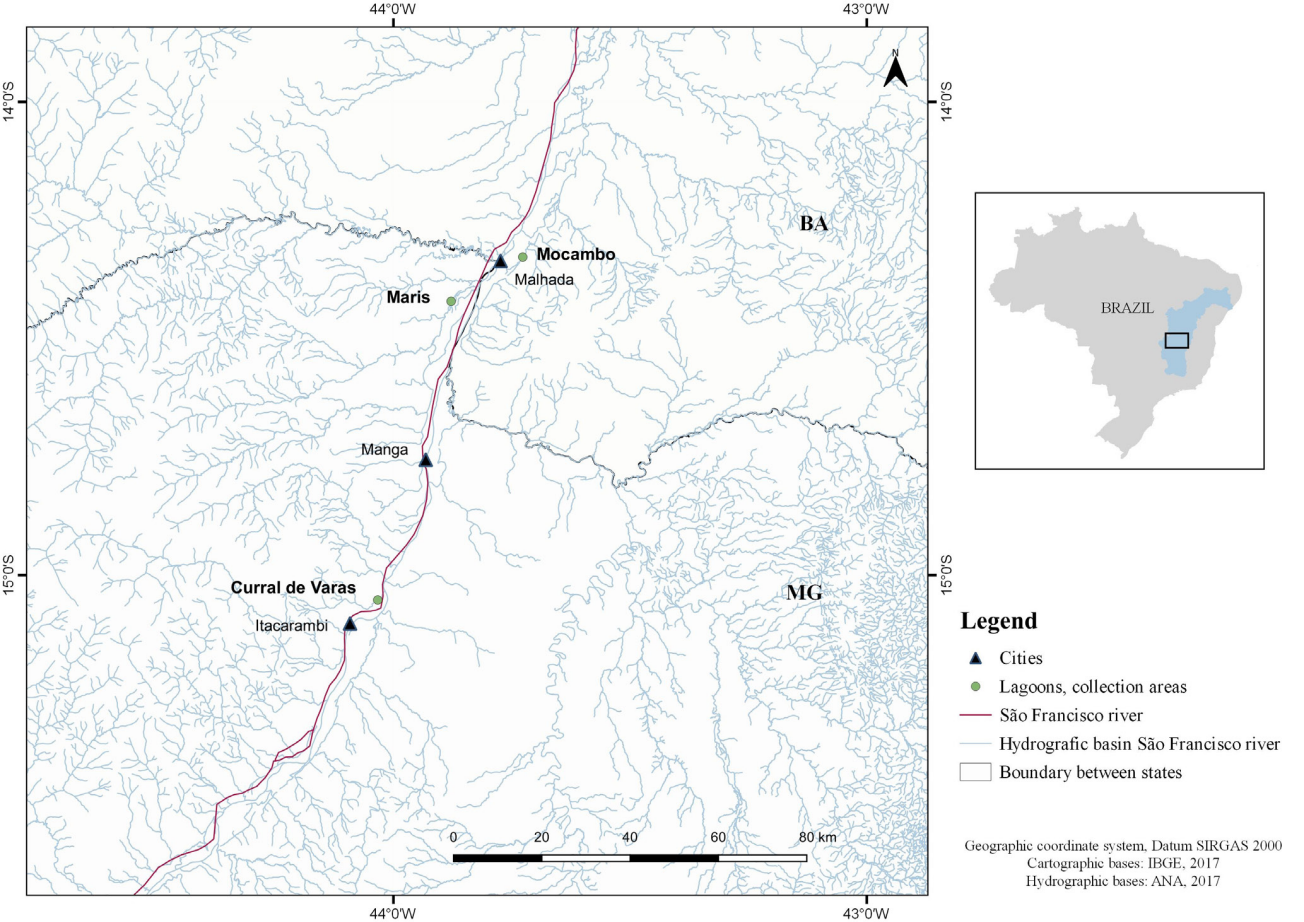
Collection areas (lagoons) for *Pygocentrus piraya* in the region of the middle São Francisco River basin, states of Minas Gerais (MG) and Bahia (BA), Brazil.

Among the 80 specimens of *P. piraya*, 53 specimens came from three marginal lagoons of the upper São Francisco River basin: 17 specimens (ten males and seven females; with mean total length (MTL) of 22.0, ranging from 17.0 to 28.0) were collected from Curral de Varas lagoon (15º03'09" S; 44º02'00" W), on the left bank of the River, in the municipality of Itacarambi, state of Minas Gerais, in October 2007; 15 specimens (five males and ten females; MTL 18.5, ranging from 14.0 to 23.0) were collected from Maris lagoon (14º25'17" S; 43º52'42" W), on the left bank of the River, in the municipality of Manga, state of Minas Gerais, in October 2008; and 21 specimens (thirteen males and eight females; MTL 26.6, ranging from 16.0 to 35.0) were collected from Mocambo lagoon (14º19'40" S; 43º43'37" W), on the right bank of the River, in the municipality of Malhada, state of Bahia, in October 2007. The QGIS 3.14.16 software was used in conjunction with the GRASS 7.8.3 software to produce maps (Figure 1).

Some better-developed nymphs were removed from their cysts and mounted on temporary slides in Hoyer medium or mounted in Amann lactophenol for examination and identification and were later on stored in 70º GL ethanol. Some specimens were imaged and measured under an Opton optical microscope coupled to a camera, using the DinoCapture 2.0 software, version 1.3.5. These measurements were presented as micrometers (μm) or, if in millimeters, this was indicated just after the value. The presentation of morphometry measurements (in the results) followed Giesen et al. (2013) and Vicentin et al. (2013).

Taxon identification at genus level and subsequent classification followed Self (1969) and Christoffersen & De Assis, (2013), respectively. The nomenclature of the taxa was as described by Poore (2012). However, the endings of species names used in the literature present variations, such as -is or -*e* in *gracilis*/*gracile*, currently used in combination with *Leiperia*, and -um or -*a* in *oxycephalum*/*oxycephala*, currently used combination with *Sebekia*. Given this doubt (i.e. whether they were proposed and treated as a noun or an adjective in the original binomials), the spelling of the names was maintained as cited by the authors of the articles consulted.

Voucher specimen of *Sebekia* sp. from *P. piraya* were deposited in the Helminthological Collection of the Oswaldo Cruz Institute (Coleção Helmintológica do Instituto Oswaldo Cruz, CHIOC), Rio de Janeiro, RJ, Brazil, under the number CHIOC 39330 (Maris lagoon). The fish voucher specimen was deposited in the Zoological Museum of the University of São Paulo, São Paulo, SP, Brazil, under the number MZUSP 95149. The ecological descriptors used were in accordance with Bush et al. (1997).

## Results

Sebekidae Sambon, 1922

*Sebekia* Sambon, 1922

**Figure 2:**
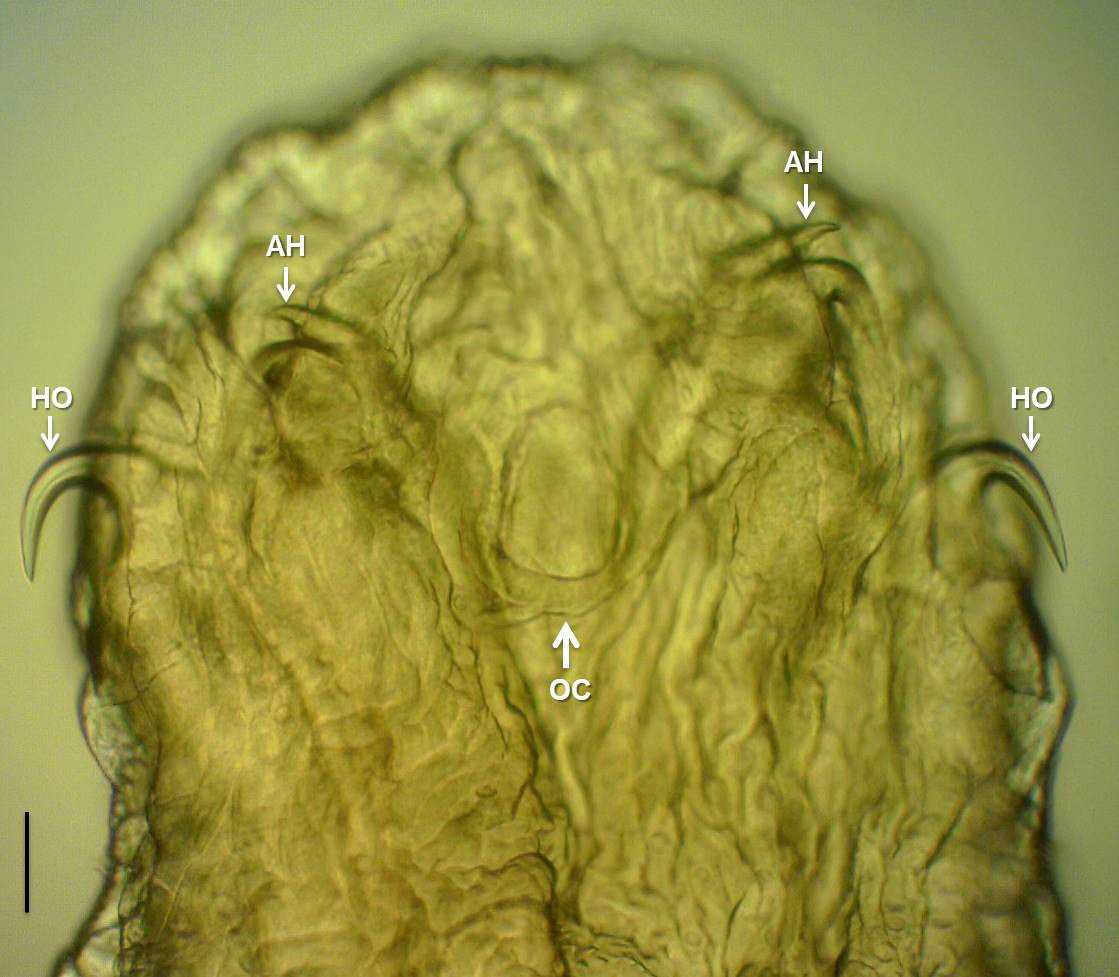
Anterior end of *Sebekia* sp. parasite of *Pygocentrus piraya* from Maris lagoon, middle São Francisco river basin, state of Minas Gerais (MG), Brazil. Nymphal specimen clarified with Amann's lactophenol, arrows HO = hooks, arrows AH = accessory hooks, arrow OC = oral cadre, scale = 50 μm.

*Sebekia* sp. (Figure 2)

Based on seven nymphal specimens (removed from the cyst): Body with anterior end rounded and tapering slightly at the posterior end, 5.2 mm (3.76 - 7.31) in length, 1.09 mm (0.86 - 1.45) in width, with 50 or 55 distinct annuli with spines on their edges; chloride cell pores large, arranged in single row at anterior border of each annulus, except closest to the anterior end, which may be arranged in two (or three) or more rows midway between annuli; anterior end with two pairs of double hooks with fine ends, each with a principal hook and another smaller accessory (Figure 2); anterior pair of hooks with principal hook 124.5 (85.15 - 154.29) in length, base 91.37 (69.41 - 106.53), blade gap 42.92 (21.96 - 56.41), fulcrum 268.53 (210.24 - 400.94) in length; posterior pair of hooks with principal hook 126.33 (94.08 - 169-80) in length, base 91.98 (69.90 - 104.35), gap of blade 41.69 (28.88 - 69.50), fulcrum 274.16 (153.48 - 382.00) in length; anterior and posterior pair of accessory hooks 102.08 (83.06 - 130.74), 96.86 (80.86 - 117.90) length, respectively; oral cadre oval in shape, chitinized, 143.6 (n = 5; 134.68 - 163.39) in length, 213.3 (n = 4; 185.31 - 233.68) in overall length, 86.4 (n = 5; 79.87 - 95.91) in width, located between pairs of hooks; anus at posterior end.

### Remarks

The specimens of the present study had general measurements greater than those presented by Giesen et al. (2013) for *Sebekia* sp. from *P. nattereri* of the Miranda River, Pantanal. In relation to the measurements of *S. oxycephala* from *P. nattereri* of the Negro River, Pantanal, the length, width and quantity of rings in the body were similar, but the measurements of the other structures were greater in the present study.

All the nymphs presented two pairs of double hooks. The anterior and posterior hooks had thin tips and bases presenting a chitinous extension from the fulcrum that formed the accessory hook. These were more robust than in the previous studies cited.

Among the 53 piranhas, from the middle section of the river that were examined, 12 were parasitized by *Sebekia* sp.. These comprised two of the 17 collected from Curral de Varas lagoon, seven of the 15 collected from Maris lagoon and three of the 21 collected from Mocambo lagoon. Table 1 presents the ecological indexes of *Sebekia* sp. per collection site; the specimens were found inside cysts in the celom cavity and surrounding organs such as the intestinal ceca and inside the fat of the hosts in the three lagoons.

**Table 1:** Occurrence of *Sebekia* sp., their parasite indexes (prevalence - P; mean intensity - MI; mean abundance - MA; standard deviation - SD), in abdominal cavity of *Pygocentrus piraya* from lagoons bordering the middle São Francisco River basin, states of Minas Gerais (MG) and Bahia (BA), Brazil.

**Pentastomid specie**	**Indexes**			**Localities (lagoons)**
	**P (%)**	**MI ± SD**	**MA ± SD**	
**Sebekidae**				
*Sebekia* sp.	11.8	1.50 ± 0.71	0.18 ± 0.53	Curral de Varas
	46.7	1.00	0.47 ± 0.52	Maris
	14.3	7.33 ± 9.29	1.05 ± 3.94	Mocambo

## Discussion

In the adult stage, sebekid pentastomids are parasites of the respiratory tract of vertebrates (definitive hosts), mainly crocodilian reptiles (Junker & Boomker, 2006; Brito et al., 2012). These parasites lay eggs containing larvae in the lungs of their definitive hosts. These are then coughed up and swallowed, such that they pass through the gastrointestinal tract and are deposited in water through feces. These eggs containing larvae are then ingested by intermediate hosts such as fish, where they hatch. The larvae that emerge develop through several molts into nymphs. When the intermediate host is consumed by the final host, the endoparasite pierces the intestinal wall and migrates to the lungs (Paré, 2008; Christoffersen & De Assis, 2015).

Christoffersen & De Assis (2013, 2015) listed the various natural intermediate and definitive hosts of pentastomids and cited the cases of visceral pentastomiasis in humans that had been in the literature. Humans can also become infected through ingestion of eggs contained in respiratory secretions, blood, saliva or feces of the definitive hosts. Presence of nymphs inside cysts in fish that form intermediate hosts in the life cycle of sebekid pentastomids may increase the possibility that the larvae may have access to humans, even accidentally (Fain, 1975; Mairena et al., 1989). Visceral parasitism by nymphs, as well as by adult pentastomids in pets and/or domesticated animals, is of public health importance (Brookins et al., 2009; Shamsi et al., 2017). From the data of the present study, we emphasize that these lagoons provide varied biotic interactions, and they are often directly accessible to animals in the interior of the states of Minas Gerais and Bahia.

Among sebekids, *Sebekia* present the highest diversity of species, with the largest spectrum of hosts, including crocodilian hosts, and the widest geographical distribution (Christoffersen & De Assis, 2015).

In Brazil, nymphs of *Sebekia* sp. or *S. oxycephala* have been found in several species of fish of diverse orders, such as Characiformes: in *P*. *nattereri* in the Cuiabá River (Rego & Vicente, 1988; Rego & Eiras, 1989), *P*. *nattereri* and *Serrasalmus marginatus* Valenciennes, 1837 in the Negro River, state of Mato Grosso (Vicentin et al., 2011; 2013); Siluriformes: in *Pseudoplatystoma corruscans* (Spix & Agassiz, 1829) and *Pinirampus pirinampu* (Spix & Agassiz, 1829) in the Cuiabá River, state of Mato Grosso (Rego & Vicente, 1988) and *Hemisorubim platyrhynchos* (Valenciennes, 1840) in the Baía River, state of Mato Grosso do Sul (Guidelli et al., 2003); Cyprinodontiformes: in *Phalloceros harpagos* Lucinda, 2008, in the Cambé River, state of Paraná (Almeida et al., 2010); and Gymnotiformes: in *Gymnotus* sp. from different fish farms in the Pantanal basin, state of Mato Grosso do Sul (Ventura et al., 2018).

The records listed demonstrate the nonspecificity of larval parasitism by these sebekids among intermediate hosts in lacustrine environments. Nymphs of such generalist and opportunistic species can evolve in either terrestrial or semiaquatic carnivorous vertebrates, which form definitive hosts that can use fish in their diets. Adult specimens of *S. oxycephala* have been reported in Squamata reptiles: snakes of the genus *Micrurus* Wagler, 1824, i.e. *Micrurus surinamensis* (Cuvier, 1816) (Elapidae), in the state of Mato Grosso (Ávila et al., 2013); and in the genus *Helicops* Wagler, 1828, i.e. *Helicops leopardinus* (as *Helicops leopardina*] in the Pantanal, state of Mato Grosso (Rego & Vicente, 1988), and *Helicops infrataeniatus* Jan, 1865, in the state of São Paulo (Silva et al., 2015). The terrapins *Hydromedusa tectifera* Cope, 1870, and *Phrynops geoffroyanus* (Scheigger, 1812) [as *Hydraspis geoffryana* (Wagler)] (Testudines, Chelidae) form hosts for less reported sebekids and are predators of fish in rivers and streams in Brazil (Rego, 1980/81; Riley, 1986).

The endoparasitic fauna of *P. piraya* from the upper São Francisco River, in the Três Marias reservoir, Minas Gerais, was studied by Santos-Clapp et al. (2022) and no pentastomid specimens were found. The presence of *Sebekia* sp., now recorded in *P. piraya* from the same basin, but originating from marginal lagoons, indicates that the characteristics of these sites favor ingestion of sebekid eggs by piranhas, possibly due to closer contact between these intermediate hosts and crocodilians, i.e. the recognized definitive hosts.

Six species of caimans (Alligatoridae, Caimaninae) are distributed in Brazil (Santos et al., 2008). Populations of *Caiman crocodilus crocodilus* (L., 1758); *Caiman latirostris* (Daudin, 1802) and *Paleosuchus palpebrosus* (Cuvier, 1807) occur in areas of the Cerrado biome (Coutinho et al., 2013). The following have been recorded in the São Francisco River basin: *C. latirostris,* the broad-snouted caiman, and *P*. *palpebrosus,* Cuvier’s dwarf caiman (Santos et al., 2008, Filogonio et al., 2010, Coutinho et al., 2013). *Sebekia oxycephala* [as *Pentastoma oxycephalum* Diesing, 1835] was described from specimens collected from *C*. *crocodilus* [as *Caiman sclerops* (Schneider, 1801)] (Self & Rego, 1985).

As well as parasitizing caimans in the Cerrado biome, sebekid species have also been well reported in caimans in the Pantanal: in *Caiman yacare* (Daudin, 1802), the black caiman, and *Melanosuchus niger* (Spix, 1825) (Self & Rego, 1985; Rego & Eiras, 1989; Junker & Boomker, 2006; Tellez, 2015). So far, *P*. *palpebrosus and Paleosuchus trigonatus* Schneider, 1801, from the Amazon, are the only species that have not been reported as definitive hosts of pentastomids (Tellez, 2015).

The parasitism records show biotic interactions that enable occurrence of the complete heteroxenic (indirect) life cycle of sebekids in the lagoons along the length of the São Francisco basin. In the case of *Sebekia* sp. in this study, these interactions involving at least the piranhas and caimans of the lagoons, without ruling out the possibility of allochthonous hosts, such as the colubrid or elapid snakes mentioned above (Santos et al., 2008; Coutinho et al., 2013).

Considering the geomorphological characteristics of the region where the three lagoons of the middle São Francisco River where the fish were collected are located, Maris lagoon is the most exposed in relation to the vegetation around it. It is homogeneous, in less rugged terrain, with less depth and a wide floodplain, and with better fluvial flow (intake). However, it is the lagoon that also dries up more easily. This set of characteristics potentially improves access for predatory organisms (including allochthonous hosts). Thus, in comparison with Curral de Varas and Mocambo lagoons, Maris lagoon was the one with the lowest number of hosts collected (15 specimens of *P. piraya*) and the largest number of fish infected by *Sebekia* sp. (seven piranhas).

## Conclusion

The results from this study form part of a project on carnivorous fish species in lagoons in the upper and middle São Francisco River. However, so far, specimens of pentastomids have only been collected from *P. piraya* in the middle stretch of the basin. Thus, the records of *Sebekia* sp. in *P. piraya* in the marginal lagoons of Curral de Varas, and Maris, both in the state of Minas Gerais and Mocambo in the state of Bahia, in the middle São Francisco River basin are novel.
